# Supplementary material for: A flexible adhesive surface electrode array capable of cervical electroneurography during a sequential autonomic stress challenge
Source: Sci Rep. 2022 Nov 14;12:19467. doi: 10.1038/s41598-022-21817-w (PMC9663551; doi:10.1038/s41598-022-21817-w)
Supplement: Supplementary file 1 — Supplementary Information. [file 41598_2022_21817_MOESM1_ESM.docx]

**Supplementary**

**Supplemental Figure 1. Heart rate comparison between pre and post timed respiratory challenge for CPTd and CPTi groups. (a)** The CPTi group had a significant change in HR after timed respiratory challenge (p<.05), whereas CPTd did not show a significant difference. Between group (CPTd vs CPTi) we observed a significant difference (p<.05) in HR change pre- to post timed respiratory challenge (p<.05). **(b) The comparison of average firing rate change with respect to baseline activity between CPTd and CPTi group across CPT intervals.** (Top Right) Rostral Channel #1 overlying the nodose ganglion and in close approximation to the auriculotemporal nerve only demonstrated a significant difference between two groups at the first two intervals, i.e., 0~40 % interval. (Bottom Right) Rostral Channel #2 overlying the nodose ganglion and in close approximation to the auriculotemporal nerve showed significant difference between two groups at 0~40% interval and 60~80% interval. (Left Panels): Caudal Channel #3 and Caudal Channel #4, overlying the carotid artery, vagus nerve, sympathetic chain and sensory C2/C3 dermatomal nerves, showed significant difference between CPTd and CPTi groups across the whole CPT challenge. *=p<.05

**Supplemental Figure 2. The average firing rate across the 5-minute CPT experiment between CPTd and CPTi groups along with the fitted line regressed by the generalized estimating equations (GEE). (Right panels)** Rostral Channel #1 and Rostral Channel #2 overlying nodose ganglion and in close approximation to the auriculotemporal nerve **(Left Panels)**: Caudal Channel #3 and Caudal Channel #4, overlying the carotid artery, vagus nerve, sympathetic chain and sensory C2/C3 dermatomal nerves. **δ**: significant trend level (p<0.1), **δδ**: significant trend level (p<0.06).

**Supplemental Figure 3. (a) Sternocleidomastoid (SCM) activity in the CPT test.** Left Panel: SCM activity ratio during CPT between CPTd and CPTi group. Right Panel: SCM activity firing rate between CPTd and CPTi group. **(b) Pre to post deep breathing change.**  Left panel: Pre to post breathing cycle change among all subjects.

Right Panel: Pre to post breathing cycle change between CPTd and CPTi group. No significant difference was found in all comparisons.

**Supplemental Table 1. The fixed and the interaction effects of group and time on firing frequency from the generalized estimating equation.** Group: CPTd vs. CPTi. Time: Time in second as a variable over the entire 5-minutes session. Sec50plus: Inflection time point at 50 second. Sec200plus: Inflection time point at 200 s. There were significant CPT-group-by-time interaction effects from 0 to 50 seconds at Rostral Channel #2, Caudal Channel #3 and Caudal Channel #4, and significant CPT-group-by-50-second-inflection-time interaction effects across all four channels (p<0.1).

**Supplemental Table 2. Summary result of the group (CPTd vs. CPTi) effect on pre-to-post respiration firing frequency change from the generalized estimating equation.** All four channels showed significant group effect difference (p<0.02).
